# The implementation of telemedicine in wound care: a qualitative study of nurses’ and patients’ experiences

**DOI:** 10.1186/s12913-024-11620-w

**Published:** 2024-09-29

**Authors:** Kjersti Marie Blytt, Beate-Christin Hope Kolltveit, Marit Graue, Mari Robberstad, Thomas Ternowitz, Siri Carlsen, Marjolein Memelink Iversen

**Affiliations:** 1 Department of Health and Caring Sciences, Western Norway University of Applied Sciences, P.O. Box 7030, Bergen, 5020 Norway; 2 Department of Medicine, Section of Endocrinology, Stavanger University Hospital, Stavanger, Norway; 3 Department for Dermatology, Stavanger University Hospital, Stavanger, Norway

**Keywords:** Telemedicine, Wound care, Foot ulcers, Diabetes, Interview, Thematic analysis

## Abstract

**Background:**

The increasing use of telemedicine (TM) represents a major shift for health workers and patients alike. Thus, there is a need for more knowledge on how these interventions work and are implemented. We conducted a qualitative process-evaluation alongside a larger randomized controlled trial designed to evaluate a telemedicine follow-up intervention for patients with a leg- or foot-ulcer, who either have or do not have diabetes. Accordingly, the aim of this study was to explore how both health care professionals and patients experienced the implementation of TM follow-up in primary care.

**Methods:**

The intervention comprised an interactive TM platform facilitating guidance and counselling regarding wound care between nurses in primary care and nurses in specialist health care in Norway. Nurses and patients from seven clusters in the intervention arm were included in the study. We conducted 26 individual interviews (14 patients and 12 nurses) in primary care between December 2021 and March 2022. Thematic analyses were conducted.

**Results:**

The analyses revealed the following themes: (1) enhancing professional self-efficacy for wound care, (2) a need to redesign the approach to implementing TM technology and (3) challenging to facilitate behavioral changes in relation to preventive care. As to patients’ experiences with taking part in the intervention, we found the following three themes: (1) experience with TM promotes a feeling of security over time, (2) patients’ preferences and individual needs on user participation in TM are not met, and (3) experiencing limited focus on prevention of re-ulceration.

**Conclusions:**

TM presents both opportunities and challenges. Future implementation should focus on providing nurses with improved technological equipment and work on how to facilitate the use of TM in regular practice in order to fully capitalize on this new technology. Future TM interventions need to tailor the level of information and integrate a more systematic approach for working with preventive strategies.

**Clinical trial registration:**

NCT01710774. Registration Date 2012-10-17.

**Supplementary Information:**

The online version contains supplementary material available at 10.1186/s12913-024-11620-w.

## Background

Telemedicine (TM) has been introduced as a decision support system for delivering follow-up care to patients with foot ulcers [1–4]. TM refers to the use of telecommunication technologies to provide clinical services to patients in a manner that improves the quality and continuity of individual treatments [4]. TM gives the opportunity for interactions at a distance between healthcare professionals in primary and specialized care [5]. The concept of TM has been developed alongside the development of different digital technologies (e.g. smartphones, wearable devices, clinical and remote sensors), which has given the opportunity to provide remote clinical services [2]. Prior studies indicate beneficial outcomes after digital health interventions in relation to smoking termination [6], increased physical activity [7], reduced blood pressure [8] and weight loss [9]. These studies are indicative of applying TM approach in this context. More recently, TM has become a more common practice in healthcare, especially in developed countries [2]. TM follow-up has the potential to improve the quality and continuity of care, reduce the need for the patient to visit the hospital and it may be more cost-effective than standard treatments [10].

Previous research has provided knowledge on the efficacy of TM treatment relative to standard outpatient care on outcomes such as ulcer healing time, amputation [1, 11], health, well-being and quality of life [3]. Furthermore, TM has been found to provide community healthcare professionals with more knowledge, improved wound assessment capabilities and more confidence [12]. However, TM applications were easier to implement at hospital outpatient clinics compared to home-based care settings [13]. Central to this understanding was that outpatient clinics at the hospital provided a work setting that eased TM, whereas the environment and work setting in home-based care was found to challenge the implementation of TM. By extension, home-based care required more individual effort from each nurse to act in a way that produced the intended high quality of care [13]. Time and adequate equipment have been suggested as central factors to take advantage of TM and to accommodate these challenges [13].

A systematic review by Hazenberg et al. [10] aimed to explore the different available TM applications that may be valuable in assessment/monitoring, prevention and/or treatment in diabetic wound care. Although several of these applications were found to be valuable, there is still need for more investigation into their effectiveness and/or feasibility [10]. Furthermore, the results from a systematic review of qualitative studies on patients, carers and healthcare professionals’ perceptions of barriers and facilitators and the use of digital technology in the management of diabetic foot ulcers, shed light on different factors that might be central to the implementation of TM [2]. The review highlights that patients’ preferences, attitudes and circumstances, healthcare professionals’ training and the support given by the organization, are all important factors to increase the probability for successful adaptation of TM. Examples of concrete barriers to the use of TM in wound care include lack of wound care competency among home care nurses [14] and unsystematic wound care training [11]. In addition, lack of interest in using digital technologies [14] and ambivalent attitudes towards the usefulness of daily wound image taking in diabetic foot care [15] are central barriers. Although several studies have investigated various aspects related to implementation, TM is still in its early stages [10]. No prior studies have investigated the wider implementation process of TM from the view of both nurses and patients in light of assessment/monitoring, prevention and/or ulcer treatment. Thus, the aim of this study was to identify how nurses and patients experienced the implementation of telemedicine in wound care.

## Methods

This study was conducted as part of a broader noninferiority parallel-cluster clinical trial among diabetes foot ulcer patients in 2013–2016 and among patients with foot or leg ulcers who either have or do not have diabetes in 2019–2021 (Clinical trial reg. no. NCT01710774). We performed embedded qualitative interviews in both trial waves to gain insights about patients’ and nurses’ perceptions and experiences of implementing the TM intervention in wound care. The results from the qualitative studies in the first wave have been published elsewhere [5, 12–14].

### Intervention

The content of the intervention has previously been reported more in-depth by Smith-Strøm et al. [1]. The TM intervention consisted of a smartphone and a web-based ulcer record. The system was developed to optimize the interaction between community nurses in primary care and specialized health personnel working at the hospital outpatient clinic. The same equipment was used by both groups. During the intervention period, the healthcare professionals documented in both an internal system and in the web-based ulcer record. The system was accessed online from both smartphones and computers. Through these applications, images and written reports and different measurements of the foot ulcer to support the imaging of the foot ulcer were accessible for all the healthcare professionals included in the intervention. The current TM intervention is somewhat different from the usual TM that most often link patients (as actual users of the technology) together with healthcare professionals. The current intervention may rather be viewed as a telemedicine platform to facilitate communication between healthcare professionals working at the hospital and community care nurses managing wound care among patients at home. Every six weeks, the patient visited the outpatient clinic for consultation. The procedure of TM follow-up care was given until an end point (healing of the ulcer (intact skin) or amputation) occurred. All the nurses in the intervention received training regarding wound treatment, follow-up and how to use the TM equipment. Physicians were involved in making the wound care procedure and they were also contacted if it was considered necessary. For each patient, there was an individual plan and if any changes occurred or if there was a deterioration of the wound, the nurses were instructed to act. The study team did however not include a care quality improvement team, implementation framework or health technologies experts, which could have been beneficial for the implementation process. However, the training that the community nurses received was a combination of standardized training and individual teaching at the specialist clinic to ensure comparable and competent handling of patients. Organizational flexibility in the municipalities was somewhat limited because of the complex nature of the intervention.

### Participants

To conduct embedded qualitative interviews in the second wave, we invited a purposive sample of 16 patients from seven of the 11 clusters. We included patients with different age, sex and functional level in order to maximize diversity. Likewise, we included nurses of different sex, age and work experience in order to reflect the group’s different characteristics that may influence their experiences. The clusters were of varying size and geographical location. Patients were eligible for participation if they were currently receiving or had recently finished the TM intervention. Nurses were eligible if they were involved or had recently been involved in the TM intervention. The term saturation is mentioned as a criterion for sample size in some qualitative studies and a central element in grounded theory. To develop new knowledge, we kept in mind the aim of the study. Thus, at the point when we considered our collected data not to give us more information regarding the aim, we discontinued our data collection. The power in the information we had gathered after these interviews indicated that we could provide new insights to respond to the research question [16]. Twelve nurses (graduated between 1993 and 2021) were invited to partake in the interviews, and all consented to participate. Eight of the nurses had additional education in wound care. Thus, a total of 26 individual interviews were conducted among patients and nurses between December 2021 and March 2022.

### Data collection

Data was collected in individual telephone interviews. The interviews had a duration of approximately 30–60 min. We have no information whether the caregivers or any others were present in the same room when the telephone interview was conducted. The interviews with the nurses included questions related to how they experienced the changes that came with TM from a nursing and organizational perspective, their perceptions of how nurses experienced their communication with the patients during the TM intervention, how they involved the patients in the treatment, and how they experienced that TM gave an opportunity to apply preventive care. The interview guide for the patients included questions regarding communication with the nurses, their own involvement during the intervention, the organization of the service, and the overall experience with the treatment process. All interviews were conducted by the second author (BCHK) and audio-recorded with the permission of the participants.

### Analysis

The recordings were transcribed verbatim. Transcripts were analyzed using Braun and Clarke’s [17, 18] thematic analysis, which provides a systematic procedure for analyzing qualitative data [19]. Thematic analysis can be used to reflect reality and themes can be identified in one of two primary ways – in a theoretical-deductive manner or using an inductive approach [17]. In the present study, we used an inductive approach, which implies that the identified themes are clearly linked to the data.

We applied a step-by-step guide in the analysis process, which involved six phases: (1) familiarizing with the data, (2) generating initial codes, (3) searching for patterns, (4) proposing and reviewing themes, (5) defining and naming themes, and (6) producing the report [17]. In phase 1, the material was collected by one of the authors. In the following process, authors KMB, BKHK, MGR and MIV read the material several times. We all aimed to read the data in an active way, which implies searching for meaning, patterns in the data and so on. As recommended by Braun and Clarke [17], we all read through the entire dataset before we started coding. In phase 2, the first author generated a list of possible codes and by doing so categorized the data into meaningful groups. The codes were discussed in the group, and further elaborated into meaningful patterns in line with our understanding. In phase 3, the first author read all the codes, went back to the original material and sorted the different codes into potential themes using mind-maps. In phase 4, we read the collected extract for each theme and discussed if they were coherent with the theme. In this process, we found that some of the data extracts did not fit together, and that we therefore needed to rework the theme. In phase 5, we defined, discussed and considered and named the themes. Phase 6 entailed the writing of this manuscript. The interviews were conducted in Norwegian, while our analyses were conducted such that we also proposed English translations of themes that were proposed and reviewed.

We have attempted to achieve trustworthiness throughout the study in terms of credibility, dependability, confirmability and transferability [20]. Three researchers with knowledge of both thematic analysis and diabetes have been involved throughout the study (BKHK, MG, MIV). BKHK is a diabetes specialist nurse and has been involved in all stages of the research project. Her experience represents a factor that might threaten reflexivity. On the other hand, substantial knowledge from the field can be a valuable source of relevant and specific research [21]. BKHK clinical experience can be seen as an advantage in the process of formulating relevant questions for the interview guide. MG is a professor in nursing with extensive methodological knowledge of thematic analyses, and MIV, MR, SC and TT have been involved in the TM intervention. Awareness of the potential influence of previous experiences has been considered in the interpretation of the data. We have sought clarity in the description of the data and the descriptions have been discussed in depth within the research team to strengthen the credibility of the study (KMB, MIV, BKHK, MR). The interviews were all performed by BKHK, which increased the likelihood that the interview guide was used in the same way in all the interviews. To strengthen dependability and to facilitate confirmability, the systematic procedure (as described in the Method section) is clearly presented in the Result section with main themes as headings followed by quotations (KMB).

## Ethical considerations

The study was approved by the *Norwegian Agency for Shared Services in Education and Research* (Ref. 837886) and the main project was approved by *Regional Committee for Medical Research* (REK-VEST 2011/1609). Informed written consent was obtained from all participants. The written information specified that participants’ contribution was voluntary and that they could withdraw from the study at any point without consequences.

## Results

To explore how nurses and patients experienced the implementation of TM in wound care, we organize the Results section in two parts. First, we present themes related to the community nurses’ experiences of implementing TM in primary care and using the TM communication platform. Second, we present themes related to patients’ experiences from taking part in the intervention.

The analysis revealed the following themes related to the community nurses’ experiences: *1) enhancing professional self-efficacy for wound care*,* 2*) *a need to redesign the approach to implementing TM technology*, and *3) challenging to facilitate behavioral changes in relation to preventive care.* We treat each of them in turn.

### Enhancing professional self-efficacy for wound care

The health care professionals experienced that access to the TM platform gave them a feeling of security and thereby enhanced their ability to perform better follow-up care for people with foot and/or leg ulcers. The interactive web-based ulcer record changed the nurses’ everyday work life as the platform made it possible to seek help regarding ulcer treatment from more experienced healthcare professionals in specialist healthcare. The platform also made it possible for the community nurses to receive guidance and confirmation regarding their own thoughts on the follow-up care. As a result of this, quick and easy access within healthcare services made it possible for the community nurses to act fast, correct and with greater confidence than without TM. The nurses expressed that the opportunities related to the TM technology and the support thereof was important to them. Furthermore, they believed that this interaction made the patients feel more secure. The nurses experienced that the platform made them feel as though they were working as part of a team, although they were on their own in the field. One nurse said: *“[This is a] great tool when I am insecure about how to proceed. I also get quick answers”.* Similar statements were expressed by nurses working in both rural and urban areas. However, nurses who worked in more rural districts expressed even more appreciation. One nurse said: *“I think that telemedicine has been very good and supportive for those of us who are working in the districts”.* Taken together, the nurses clearly valued the opportunity and support that TM provided. Noticeably, this was also expressed by their wish that TM would proceed as a tool in practice going forward, and that it should be available for all the nurses in their community. As stated by one of the nurses; *“[But] I hope that TM will be available again”.*

### A need to redesign the approach to implementing TM technology

The nurses claimed that TM was not well-integrated in relation to other work tasks. Therefore, they expressed a need for better organization and integration of the service. In the words of one nurse: *“It is not organized well enough. This might be my own fault. It takes a lot of time. We are doing this on top of everything else”.* Also, technical issues are mentioned as a barrier and a possible explanation for the lack of integration that the nurses experienced in the implementation process. As stated by one nurse: *“We have experienced problems with the connection to the telecommunication network because of the phone that is part of the nursing service. Several of the nurses find it difficult to follow up using telemedicine because of this issue”.* Other technical challenges were also expressed. One nurse stated: *«I found it unfavorable that I did not have my own phone with camera. I had to use a phone that I shared with my colleagues. And this led to interruption in action. I had to answer the phone and other practical tasks”.* Hence, the results indicate that there is room for further organizational and technical improvements in implementation and delivery of TM. Consequently, the role of health service managers is key. This was also expressed by the nurses as they highlighted that they did not get enough time to really get familiar with TM and consequently felt that it was not fully internalized.

### Challenging to facilitate behavioral changes in relation to preventive care

As part of the implementation of TM, the nurses were also asked how they were working with preventive care strategies in order to prevent recurrent ulcers. The data revealed that some nurses found it difficult to involve patients in preventive aspects and to produce lasting behavioral changes. A nurse explained it as follows: *“Some of the patients are interested in preventive strategies and some do not care. They don’t want to take responsibility for their own health. Some of these patients are very set in the way they are living their lives and they want us to stop nagging”*. Similar statements were expressed from other nurses. For instance, one nurse stated: *“My experience is that they are interested in doing preventive measures*,* but they find it hard to pull through”.* Some of the nurses also expressed that patients seemed interested in taking action and doing preventive measures. Furthermore, they believed that patients were interested and involved when they received information about it. Moreover, nurses expressed that they gave plentiful advice to the patients regarding different preventive measures. However, they experienced that the patients often did not follow up the plan afterwards. Thus, the nurses found it challenging to implement preventive care strategies and produce lasting behavioral changes.

As to patients’ experiences related to taking part in the intervention, we found the following three themes: *(1) experience with TM promotes a feeling of security over time*,* (2) patients’ preferences and individual needs on user participation in TM are not met* and, *(3) experiencing limited focus on prevention of re-ulceration.*

### Experience with TM promotes a feeling of security over time

The interaction between healthcare professionals at different levels in the health care system provided the patients with a positive experience and made them feel more secure. Hence, we found that through interaction, communication and follow up, the patients experienced a feeling of security regarding TM and its functions. As stated by one patient: “*When the wound was quite big and painful*,* I felt more insecure if it was the nurses with less competence who came to assist me. But it worked very well. Sometimes they contacted help via the phone. They have contact and receive help from the hospital and I find this reassuring. The nurses at the hospital decide which treatment and follow up should be carried out by the community nurses.”* In addition, the patients expressed that they wished that TM treatment would be continued, and they stated that it was beneficial and essential for good treatment. Taken together, the results indicate an initial worry and a sense of insecurity when less competent nurses were responsible for wound care. However, TM overcame this issue, and the patients developed a feeling of security and expressed that they hoped that TM would proceed.

### Patients’ preferences and individual needs on user participation in TM are not met

Patient preferences differed from patient to patient. This relates to how much they wished to be involved in the treatment and how much detail they would like regarding the communication that is exchanged via the TM platform. The results indicate that each patient’s different needs were not fully met in the implementation of TM. Some of the patients said they would like to be little involved and received little information. One patient said: *“I felt safe*,* I think I knew too little about this and the nurses would do what they knew were correct and best for me”.* On the other hand, some patients would like to be more involved and to receive as much information as possible. One patient stated: *“What I think seems a bit odd is that I did not get clear feedback about whether the information that was handed to the hospital on my behalf had any implications for the treatment. I never received any feedback about whether the information I provided was received or not at the hospital.”* Also, the patients pointed out the need for individual adaption in TM follow-up care. In the words of one patient: *“I am a person with quite good communication skills*,* but that is not the case for everyone in this patient group. I think they need to take it very slowly and carefully with other patients. This is something that is very new for the elderly in this patient group.”*

### Experiencing limited focus on prevention of re-ulceration

A central theme in the interviews with patients revolved around their thoughts about different preventive measures. This included perceptions of how patients themselves take preventive measures as well as how the healthcare professionals communicated the importance of this to the patients. The patients in the present study mainly reported that there was little or no focus on prevention. In the words of one patient: *“No*,* I cannot remember that any of them has talked about prevention”*. Similar statements were provided from several participants, for instance as follows: *“No*,* they have not talked about it. The only thing they have mentioned is that I should be careful not to knock my foot into a table or something like that.”* It is unclear if this is indeed the case or if the patients did not have enough knowledge to determine what constitutes preventive measures. If so, they would not be aware that nurses provide them with important easy-to-use advice that is crucial for daily management and prevention of new ulcers. However, this sheds light on an area in which there is room for further improvement regarding how such information is provided to this patient group.

## Discussion

We found that implementation of TM gave nurses an increased ability to act although there are still minor, but essential barriers to full integration. The patients expressed an increased feeling of security after having had positive experiences with TM. In addition, the patients reported that they had different preferences and needs regarding how much information they would like on what is communicated on the TM platform and how involved they wanted to be. Furthermore, the results indicate that although TM provides the community nurses with a tool to be more proactive in preventive care, the nurses found it challenging to facilitate behavioral changes in relation to such care. This is a complex issue as some of the patients report that they experience receiving limited information on strategies to prevent-re-ulceration.

The first theme describes an “enhancing professional self-efficacy for wound care”, which also implies that the knowledge and guidance provided to the community nurses are facilitating their actions. This is in line with previous research that has shown that TM increased health care professionals’ skills and knowledge on wound assessment [11, 12, 22, 23]. Increased knowledge, and consequently an enhanced ability to act, are important elements when evaluating the use of TM. Through these factors, the nurses may experience improved confidence and a feeling of security. This can in turn empower them to act and give high quality care.

These results are also reflected in the patients’ feedback, as they reported having developed more security through their experiences with TM. Furthermore, different factors such as self-esteem, positive emotions, and organizational and personal commitment have been found to mediate job satisfaction [24]. Considering this, it would be interesting to investigate how TM may affect or mediate job satisfaction, as it provides the nurses with an increased ability to act. This is important, not merely because of the positive aspect of job satisfaction in itself, but also because job satisfaction is a determinant of turnover intention. For instance, Perry et al. [25] found that turnover intention (i.e., employees’ intent to find a new job with another employer) is more present among employees that are less satisfied with their jobs. Hence, the results from the present study may have other important implications beyond the scope of this paper.

The results indicate that there are areas with room for further improvement. Although the nurses in the present study reported that they felt more secure and that TM provided them with an increased ability to act, they also recognized that TM has the potential to function even better. The nurses described different challenges with the technology that could be improved. These issues include difficulties with the basic services as the telecommunication system. Moreover, it also relates to more comprehensive issues. One such issue relates to a feeling that the application is not fully integrated in work life. Another relates to the experience of TM as a time-consuming process and consequently increasing workload, contrary to the intention of the use of TM.

The nurses expressed that the service managers should provide them with more time and resources to really get familiar with TM and thus be able to fully integrate it in practice. These results are in line with a scoping review that pointed out that the technology itself is a hindrance due to a shortage of resources and time, training, and finances [26]. This is also reflected in previous results from an earlier stage in this project showing that documentation is seen as overly time-consuming [12]. Moreover, adequate equipment and time is essential to benefit from this new technology [13]. In order to address these challenges, the responsibilities of health service managers in primary care need to be addressed more. These responsibilities also need to be specified to facilitate the adoption of new technologies and work to integrate them in practice. Consequently, the use of TM technology can be a relevant alternative and supplement to usual care [1].

The main difference between the current study and the qualitative study conducted in the first wave of the TM project [12] is that the current study provides knowledge regarding the experiences of both health care professionals and patients. Furthermore, Kolltveit et al. [12] found that introducing TM in primary and specialist health care implied a change in wound assessment knowledge and skills. The authors further emphasized that the health care professionals developed a better and increased understanding of what they saw when they evaluated the ulcers and performed wound care. The results from the present study indicate a change in relation to the nurses’ everyday work life, as the online web ulcer platform made it possible to seek help regarding the ulcer treatment from more experienced healthcare professionals.

The results further highlight that the differences in patient preferences and needs are central to consider in the implementation of TM. Some of the patients express that they would like to be more involved, while others express that they do not want further information or involvement. The latter group would therefore like the nurses to take full control over their situation. Two central concepts in the health science literature are person-centered-care or patient-centered care, respectively [27]. Both concepts imply that patients should be more involved as partners in their care and treatment. Thus, they highlight that the patient should be the most central stakeholder in the decision-making process [28]. A recent systematic review investigating the use of person-centered care in chronic wound care found improved outcomes regarding pressure ulcer prevention, patient satisfaction, patients’ knowledge and quality of life [29]. Considering the findings from Gethin et al. [29] and the results from the present study, we argue that patients’ needs, beliefs, strengths and personality should be carefully considered in the implementation process. This is also highlighted and pointed out by Foong et al. [2], who argued that patients’ preferences, attitudes and circumstances are important factors for successful adoption of digital technology in diabetic foot ulcer treatment.

The results showed that nurses found it challenging to facilitate behavioral changes in relation to preventive care. This is also somewhat reflected in the answers from patients, as they express that the nurses have limited focus on different strategies to prevent re-ulceration. However, the difficulty of overcoming patient inertia for facilitating change in behavior may not be directly linked to TM use. Instead, it may reflect that the nurses are not truly integrating technology into the process of care. This would require more careful planning and resourcing of the technology implementation process. Meanwhile, the nurses report working with preventive care – communicating central and easy-to-use advice – but experiencing that the patients do not make use of this information. To understand the discrepancies in how these experiences from nurses and patients are intertwined goes beyond the scope of the present study. However, it points out central aspects for future studies to examine. Moreover, we found that different patients had different needs and preferences – an aspect that could also be further improved through patient-centered care. For instance, Li et al. [30] found that an individualized, educational 12-week program (including one-on-one training during bedside visits, brochures, telephone follow-ups and home visits) significantly improved foot self-care behavior. However, there was no change regarding the incidence of the foot problems. Considering this study and the results from the present study, we argue that future implementation of TM should consider even more systematic and individual-level work with preventive care strategies. TM provides nurses with a tool to be more proactive in preventive care, but it seems difficult to overcome these challenges by only implementing TM without a complimentary behavioral strategy.

Treatment of foot ulcers among people with and without diabetes challenges the health care system in terms of resource distribution and management strategy [31]. By extension it has been important to investigate the effectiveness of TM, as it may represent an approach that can meet these challenges. The quantitative results that the current study derives from was reported in Smith-Strøm et al. [1] and Iversen et al. [3]. Smith-Strøm et al. [1] found that TM follow-up to patients was noninferior for ulcer healing time when compared with standard outpatient care. Furthermore, when comparing risk from death and amputation was considered, no significant differences in healing time was found. In secondary analyses of data from this study, Iversen et al. [3] aimed to compare changes in self-reported health, well-being and quality of life. The findings showed no significant differences between the intervention and control group in changes in scores for the patients’ reported outcomes. The current study found some room for further development in how we integrate TM in regular practice and need for better technological equipment. However, we do not know how patients experiences standard care and potential challenges that arise in such care compared to those in the TM setting. The current study does still provide essential insight that enriches our understanding of TM as an appropriate and effective method for wound care management and treatment.

### Study limitations

The insights from this study provide knowledge on how nurses and patients experience the implementation of TM in primary care. A potential limitation is that the answers from patients might have been influenced by the interactions and established relationships with the nurses. This may have led them to give more positive answers to show gratitude for the treatment and their involvement. In addition, the nurses may have cultivated a specific culture within the clusters that may have affected their answers. This could lead to responses not necessarily representing their own actual views and experiences. The interviews were conducted over the telephone, which may have led to loss of nonverbal data that could have provided contextual information that could in turn have informed the analyses. As this is a technology-based intervention, one can argue that it would have been beneficial if the interview guide included specific questions regarding technical issues with and barriers to operating the TM technology. This might have provided us with clearer and more detailed information regarding the possible barriers when using this method. In addition it would have been beneficial if this further implementation study also included interviews with the healthcare professionals working at the hospital as this may have provided us with knowledge regarding their experience with the TM intervention. Both of these issues would be a fruitful topic for further research on similar TM approaches.

## Conclusions

The results from the present study provide new and valuable knowledge on how the implementation of TM is experienced both from nurses’ and patients’ perspectives. Future implementation of TM should provide nurses with better technological equipment, mapping each patient’s specific needs for information about the communication that is exchanged on the TM platform and provide nurses with tools that facilitate a systematic approach in order to produce behavioral changes in relation to preventive care.

## Electronic supplementary material

Below is the link to the electronic supplementary material.

Supplementary Material 1.

Supplementary Material 2.

Supplementary Material 3.

## Data Availability

The datasets generated during and analyzed during the current study are not publicly available in order to ensure the participants ‘anonymity but are available from the corresponding author on reasonable request.

## Abbreviation

TM: Telemedicine
