# Supplementary material for: The implementation of telemedicine in wound care: a qualitative study of nurses’ and patients’ experiences
Source: BMC Health Serv Res. 2024 Sep 29;24:1146. doi: 10.1186/s12913-024-11620-w (PMC11439246; doi:10.1186/s12913-024-11620-w)
Supplement: Supplementary file 2 — Supplementary Material 2. [file 12913_2024_11620_MOESM2_ESM.docx]

| Interview guide - patients |  |
| --- | --- |
| Theme | Main questions |
| Opening. | Can you tell a bit about yourself and your situation with foot ulcers? |
| Experience of having foot ulcers | Do you currently have foot ulcers, and how long have you had them? If you don't have foot ulcers now, how long ago were they healed? |
| Experience of the treatment process. | Can you, in a general sense, say something about your experiences with being followed up by the healthcare service (home nursing) regarding wound care treatment? |
| Participation in the treatment process. | Regarding being active in your own treatment or having the opportunity to be involved in your own treatment, what is your experience with this? Can you describe to what extent you wish to be involved in wound care treatment? |
| Communication in the treatment process. | How did you experience the communication between you and the healthcare professionals who followed up on wound care treatment in the municipality healthcare service? How did you experience the communication between the nurse in the municipality versus the nurse in the outpatient clinic? Can you describe what is perceived as similar/different |
| Collaboration in the treatment process. | How do you experience the contact/collaboration between you and the healthcare professionals who follow you up in wound treatment? |
| Organization of the service offering. | How do you perceive the organization of the healthcare service in relation to the follow-up of your foot/leg ulcer? |
| The patient's focus on wound prevention. | In terms of preventing new wounds, what reflections do you have on that? |
| Healthcare professionals' focus on wound prevention. | When it comes to the topic of preventing new wounds, do you have any thoughts or experiences on how healthcare professionals have focused on preventing new wounds? |
| Conclusion. | We are approaching the end, and I have just a few final summarizing questions:   - Can you describe what you think is the most important aspect of the treatment process you have experienced? - Is there anything related to the prevention of foot ulcers that you think we haven't discussed? - Is there anything related to involvement/participation (collaboration/communication) that you think we haven't covered, which you find important? - Is there anything I haven't brought up that you would like to talk about? |
