# Supplementary material for: The implementation of telemedicine in wound care: a qualitative study of nurses’ and patients’ experiences
Source: BMC Health Serv Res. 2024 Sep 29;24:1146. doi: 10.1186/s12913-024-11620-w (PMC11439246; doi:10.1186/s12913-024-11620-w)
Supplement: Supplementary file 3 — Supplementary Material 3. [file 12913_2024_11620_MOESM3_ESM.docx]

| Interview guide - nurses |  |
| --- | --- |
| Theme | Main questions |
| Opening | Can you tell a bit about yourself and your education and work experience? |
| Experience of having foot ulcers | When it comes to the patient's life situation regarding foot ulcers, what are your thoughts on it? |
| Experience of the treatment process | The treatment process for patients who have received telemedical follow-up for foot ulcers has likely resulted in changes for you. Can you describe what changes have occurred and how you perceive these changes? (Feel free to provide specific examples.) |
| Organization of the workday, frameworks and structure, aspects related to how the department is organized. | How do you perceive that telemedicine as a treatment form is adapted/facilitated within the department where you work? |
| Organizational aspects related to the implementation of telemedical wound follow-up. | In terms of implementing new services, such as telemedical follow-up, can you briefly describe how the implementation has taken place in your department?" |
| Participation in the treatment process. | Regarding user involvement in patient care, what thoughts/reflections do you have on it? Is user involvement something you focus on? (Feel free to provide examples.) |
| Communication in the treatment process. | During the consultation where you follow up with a patient with foot ulcers, how do you experience the communication between you and the patient? |
| Collaboration in the treatment process. | How do you experience the collaboration between you and the patient during wound treatment? |
| Healthcare professionals' focus on wound prevention. | In relation to preventing new wounds, what thoughts do you have on that? |
| Organization regarding the prevention of foot ulcers. | We previously touched on the organization of the workday... In what way do you experience, in your daily work, that measures are taken to enable you to focus on preventing new foot ulcers? |
| The patient's focus on wound prevention. | Continuing on the topic of wound prevention, do you have any thoughts or experiences regarding the focus patients have on preventing new wounds? |
| Conclusion. | We are approaching the end, but I have some final summarizing questions:   - Can you describe what you think is the most important aspect of the treatment process for foot ulcers? - Is there anything related to the prevention of foot ulcers that you think we haven't discussed? - Is there anything related to participation (collaboration/communication) that you think we haven't covered, which you find important? - Is there anything related to participation (collaboration/communication) that you think we haven't covered, which you find important? |
